# Supplementary material for: Optimization of a Method for Detecting Intracellular Sulfane Sulfur Levels and Evaluation of Reagents That Affect the Levels in Escherichia coli
Source: Antioxidants (Basel). 2022 Jun 29;11(7):1292. doi: 10.3390/antiox11071292 (PMC9311597; doi:10.3390/antiox11071292)
Supplement: Supplementary file 1 [file antioxidants-11-01292-s001.zip › antioxidants-1776325-supplementary.pdf]

Supplementary materials for

Optimization of a Method for Detecting Intracellular Sulfane Sulfur Levels and Evaluation of Reagents That Affect the Levels in *Escherichia coli*

Qiaoli Yu [1], Mingxue Ran [1], Yuqing Yang [1], Huaiwei Liu [1], Luying Xun [1,2,*] and Yongzhen Xia [1,*]

[1] State Key Laboratory of Microbial Technology, Shandong University, 72 Binhai Road,
Qingdao 266237, China; yuqiaoli@mail.sdu.edu.cn (Q.Y.); ranmx@sdu.edu.cn (M.R.);
angyuq@mail.sdu.edu.cn (Y.Y.); liuhuaiwei@email.sdu.edu.cn (H.L.)

[2] School of Molecular Biosciences, Washington State University, Pullman, WA 991647520, USA

* Correspondence: luying_xun@vetmed.wsu.edu (L.X.);
xiayongzhen2002@email.sdu.edu.cn (Y.X.);
Tel.: +1-509-335-2787 (L.X.); +86-532-58631572 (Y.X.)

Supplementary Table S1. Screening for best signal to noise ratio (SNR) of bimane-$S_2O_3^{2-}$ with the combinations of sensitivity and gain values.

Sensitivity/gain value	Intensity	SNR
low/1	4398.7±245.8	23.57±12.8
low/4	22972±3373.5	79.6±20.4
low/16	88426±1635.4	196.6±63.7
middle/1	179047±1251.2	475.8±135.2
middle/4	730083±8179.2	1397.6±197.2
middle/16	3140390±60138.0	3011.8±201.3
high/1	1705167±78638.6	1237.8±212.6
high/4	7472120±478818.5	1.1±1.0
high/16	26006890±5211677.0	0.2±0.1

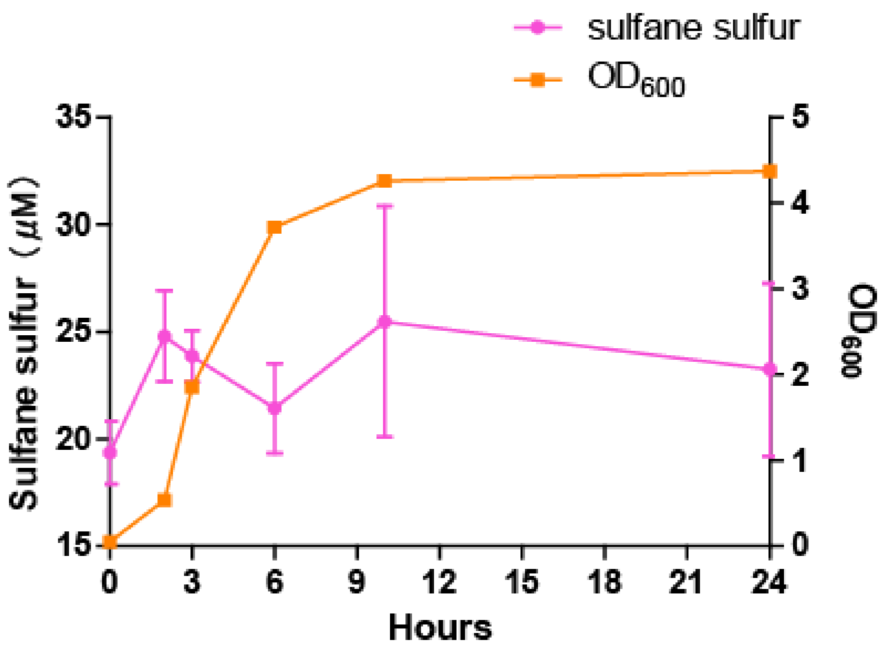

Supplementary Figure S1. Sulfane sulfur concentrations in the supernatant of *E. coli* cultures growing in LB medium. The *E. coli* BL21(DE3) cells were incubated in an LB medium at the initial OD_{600} of 0.05. With cell growth, the sulfane sulfur concentrations in the supernatants were detected with the improved SdSS method at defined time intervals. Three parallel experiments were performed to obtain the averages and standard deviations (n=3).

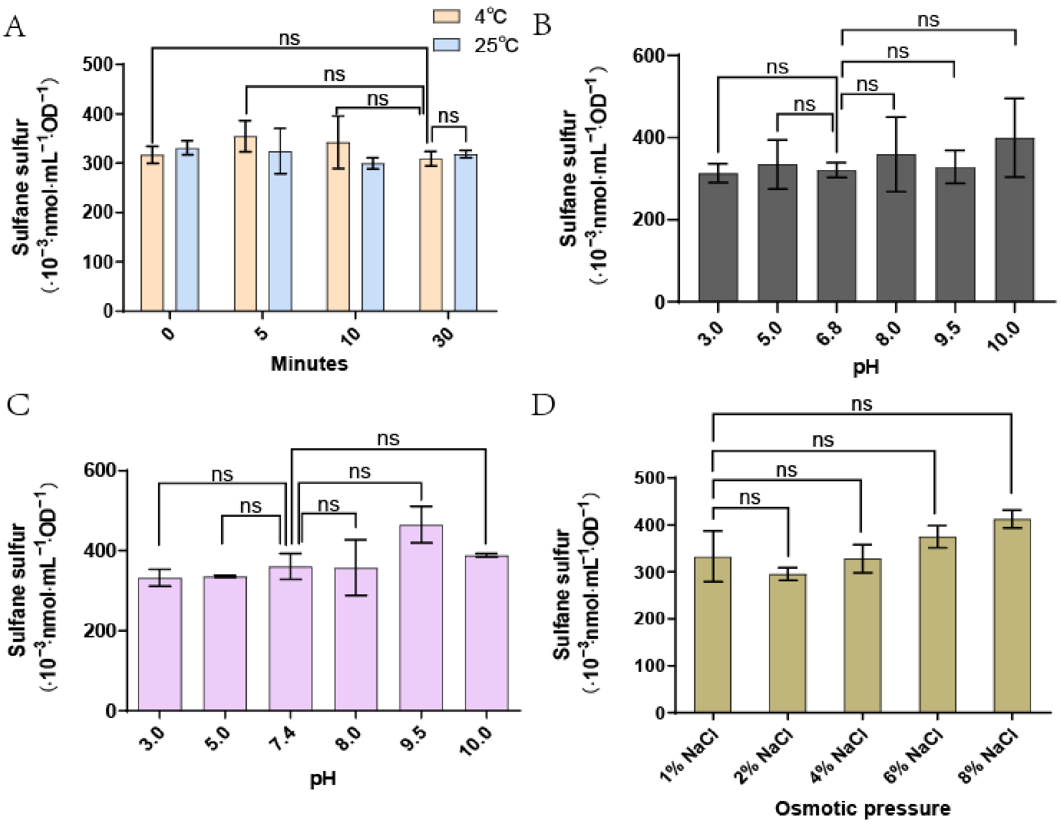

Supplementary Figure S2. The influence of temperature, pH, and osmolarity on iSS. The *E. coli* BL21(DE3) cells were cultured in LB medium until OD_{600} reached around 1.0. **A)** The cells in LB medium were incubated on ice or at room temperature for 0 to 30 min, respectively, before iSS analysis. **B&C**) The cells were collected and resuspended in LB medium **(B)** or Tris-HCl buffer **(C)** at OD_{600} of 1 with pH that was adjusted as shown in the figure. Then, cells were placed at room temperature for 10 min before iSS analysis. **D)** The cells in LB medium with different NaCl concentrations were incubated at room temperature for 30 min. After incubation, the cells were harvested and iSS contents were determined by using the optimized detection method. Three parallel experiments were performed to obtain the averages and standard deviations (n=3). The one-way ANOVA method was performed to calculate the p-values (ns, $p \geq 0.05$).

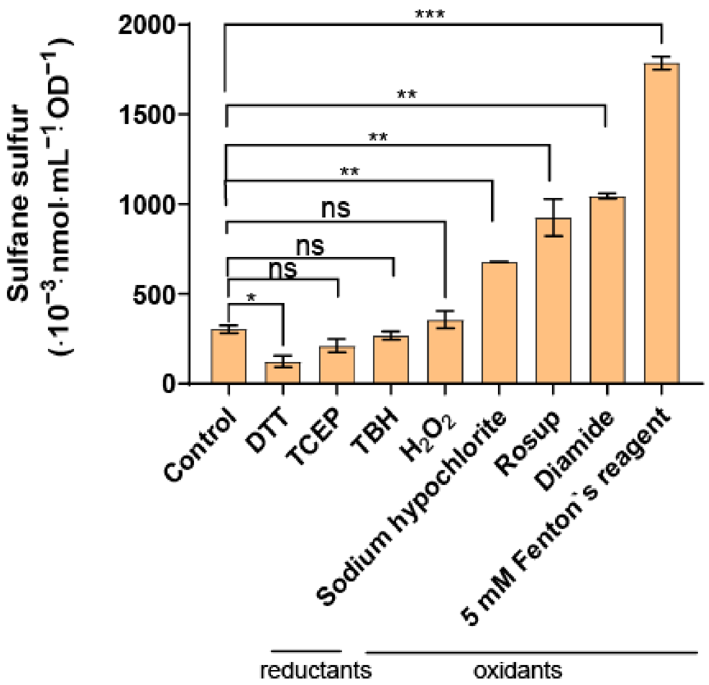

Supplementary Figure S3. Changes in the iSS content of *E. coli* cells under different oxidants. *E. coli* BL21(DE3) cells were cultured until their OD_{600} reached 1.0. Then, the cells were aliquoted, and different oxidants were added and further incubated for 30 min before iSS detection. Fenton's reagent was used at 5 mM, and all other reagents were used at 10 mM. Three parallel experiments were performed to obtain the averages and standard deviations (n=3). The One-way ANOVA method was performed to calculate the *p*-values (ns, $p \geq 0.05$; *, $p < 0.05$; **, $p < 0.01$; ***, $p < 0.001$).

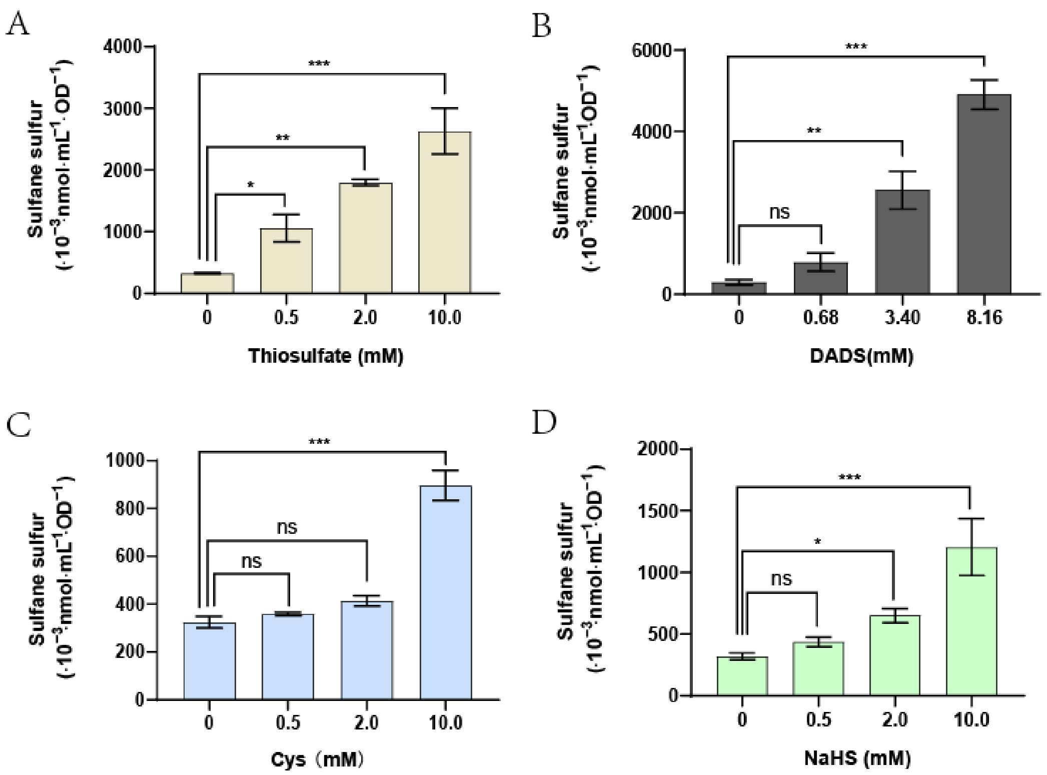

Supplementary Figure S4. The iSS contents of *E. coli* BL21 (DE3) cells with varying concentrations of sulfur-bearing compounds. Different amounts of thiosulfate **(A)**, DADS **(B)**, Cys **(C),** and NaHS **(D)** were added to the BL21(DE3) cells at OD_{600} of 1.0 in LB medium and incubated for 30 min before iSS analysis. Three parallel experiments were performed to obtain the averages and standard deviations (n=3). The one-way ANOVA method was performed to calculate the p-values (ns, $p \geq 0.05$; *, $p < 0.05$; **, $p < 0.01$; ***, $p < 0.001$).

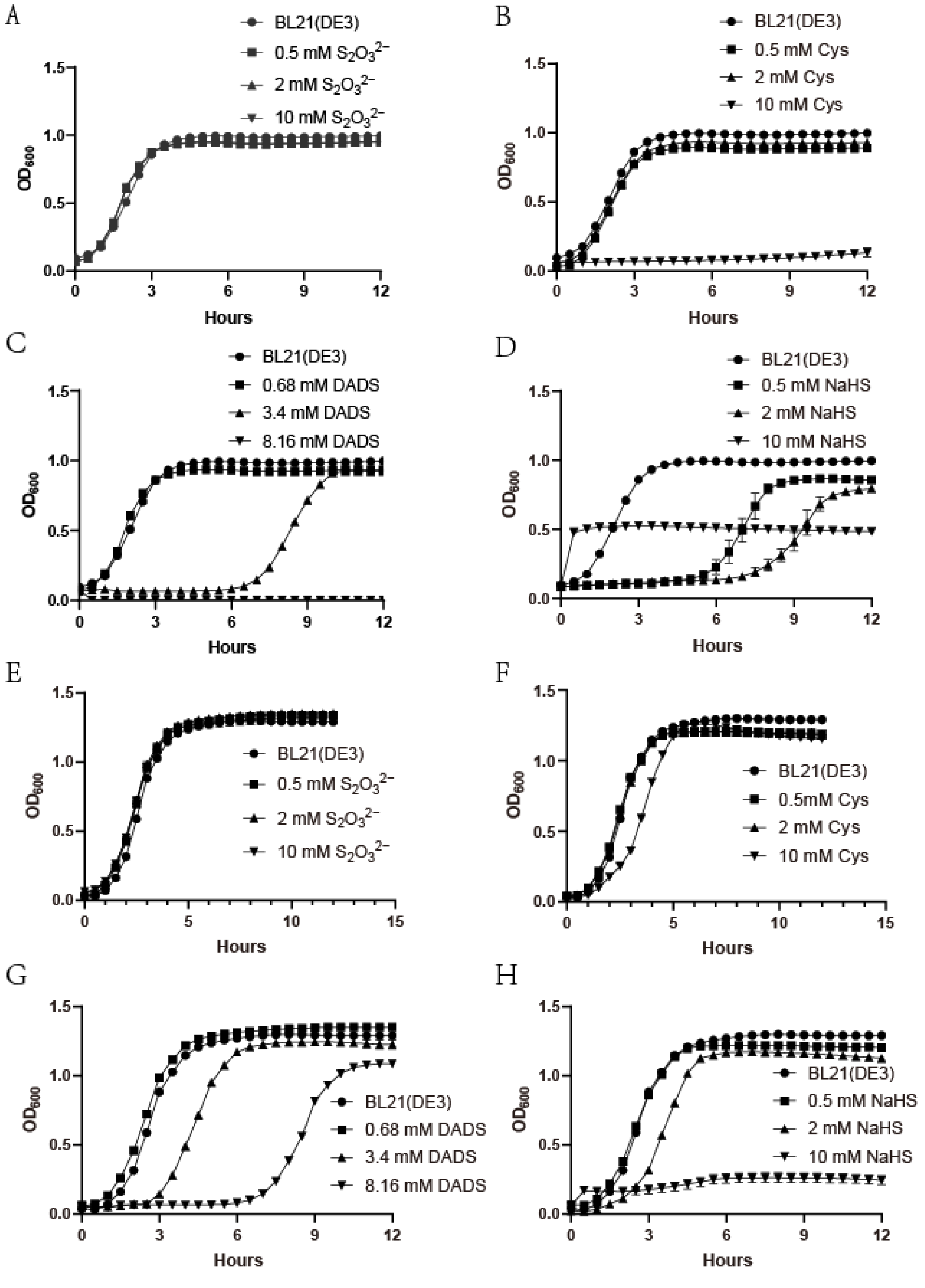

Supplementary Figure S5. Growth curves of *E. coli* BL21(DE3) when incubated with different sulfur-bearing compounds. BL21(DE3) cells were incubated in 400 μL LB medium at the initial OD_{600} of 0.05 in 48-well plates. Different sulfur compounds with defined concentrations were supplied. Then, the cultures were

incubated in a closed environment **(A-D)** and an open environment **(E-H)**. Three parallel experiments were performed to obtain the averages and standard deviations (n=3).
